# Key challenges for implementing a Canadian-based objective structured clinical examination (OSCE) in a Middle Eastern context

**Published:** 2016-12-05

**Authors:** Kyle John Wilby, Mohammad Diab

**Affiliations:** 1 College of Pharmacy, Qatar University

## Abstract

Globalization of medical education is occurring at a rapid pace and many regions of the world are adapting curricula, teaching methods, and assessment tools from established programs. In the Middle East, the use of Objective Structured Clinical Examinations (OSCEs) is rare. The College of Pharmacy at Qatar University recently partnered with the University of Toronto and the Supreme Council of Health in Qatar to adapt policies and procedures of a Canadian-based OSCE as an exit-from-degree assessment for pharmacy students in Qatar. Despite many cultural and contextual barriers, the OSCE was implemented successfully and is now an integrated component of the pharmacy curriculum. This paper aims to provide insight into the adoption and implementation process by identifying four major cultural and contextual challenges associated with OSCEs: assessment tools, standardized actors, assessor calibration, and standard setting. Proposed solutions to the challenges are also given. Findings are relevant to international programs attempting to adapt OSCEs into their contexts, as well as Canadian programs facing increasing rates of cultural diversity within student and assessor populations.

## Introduction

In 2013, the College of Pharmacy at Qatar University partnered with the University of Toronto and the Supreme Council of Health in Qatar to develop and implement a first cycle assessment for May 2014. The first cycle consisted of a 100 question case-based multiple choice exam, an open-book pharmacy practice exam consisting of developing a comprehensive patient care plan based on a paper case, and an 8-station Objective Structured Clinical Examination (OSCE). OSCEs are gold standard learning-based assessment techniques used commonly throughout health professional training programs worldwide.^1^ While OSCEs take many forms, for the purposes of this paper OSCE refers to a series of stations, each with a case or scenario that requires the learner to complete a task. OSCEs can be used for both formative and summative assessment and are used in all Canadian medical schools and for high stakes examinations, such as licensure and re-licensure, in Canada.^2^–^4^ Information obtained from OSCEs enables identification of deficiencies in candidate performance, gaps in curricula or training, and needed improvements to the assessment process.

In both 2015 and 2016, the college repeated the assessment cycle with a 10-station OSCE. The exit-from-degree OSCE is now a regular component of the undergraduate curriculum. While Western-based assessment methods are typically regarded as “best practice” throughout medical communities, we encountered many contextual and cultural considerations that threatened the validity and reliability of the OSCE as a high stakes assessment. Therefore, the objective of this paper is to report key challenges during OSCE adoption and implementation along with the associated solutions to further our understanding of transplanting assessment practices to culturally dissimilar settings.

## Methods

This paper is a descriptive report of challenges encountered when adopting and implementing the policies and procedures of a Canadian-based OSCE in Qatar. Investigator field notes were maintained throughout this process. These notes, along with data generated from OSCE cycles, were used to identify pertinent challenges relevant to the cross-cultural setting. Objective data used during this process included standard setting cut scores, candidate pass rates per station and overall, and inter-rater reliability for analytical and global assessments (calculated using 2-way random intraclass correlation coefficients [ICCs]). Qualitative data sources included 1) feedback from assessors and standardized actors via incident reporting forms, 2) candidate feedback via a debriefing session, and 3) investigator field notes. All data were analyzed by both investigators and consensus was achieved for the final challenges to be included in the paper.

### Setting

The College of Pharmacy at Qatar University hosts an entry-to-practice Bachelor of Science in Pharmacy program that is accredited by the Canadian Council for Accreditation of Pharmacy Programs (CCAPP).^5^ Students complete at least one year of general arts and sciences prior to admission to the four-year degree program. The curriculum is similar to other CCAPP accredited programs, including a 24-week experiential training program. Unlike the Canadian context, assessment for licensure in Qatar consists of only a 100 multiple choice question exam that largely assesses knowledge relating to pharmacology. As such, CCAPP recommended the College establish an exit-from-degree cumulative examination to reflect the current assessment practices implemented in Canada by the Pharmacy Examining Board of Canada.

### Cross-cultural collaboration

The collaboration between Qatar University, the Supreme Council of Health in Qatar, and the University of Toronto was formed via a consulting agreement with faculty from the Leslie Dan Faculty of Pharmacy in Toronto. Once the agreement was finalized, four faculty members from Qatar University (Associate Dean of Academic Affairs, Assistant Dean of Student and Faculty Affairs, and two Assistant Professors of Clinical Pharmacy and Practice) traveled to Canada to observe OSCE development and implementation processes with the University of Toronto and the Ontario College of Pharmacists. This visit also provided the opportunity to blueprint the OSCE according to measurable skills, such as making therapeutic recommendations, referrals, patient self-selection, adverse effect management, drug interaction management, patient counselling, healthcare profession education, and cross-cultural communication. Subsequently, the consulting faculty from Toronto made three return trips to Qatar to 1) lead case development and validation, 2) complete standardized actor and assessor training, and 3) observe the first OSCE cycle. After completion of the OSCE event, these faculty members remained available to personnel in Qatar for assistance with data analysis and evaluation. Qatar faculty members were trained during each stage to ensure sustainability in future cycles.

## Results

Four major challenges were identified that were deemed pertinent to cultural and contextual factors in our setting. All four challenges were agreed upon by both investigators and are included in this report.

### Challenge #1 – Assessment Tools

OSCE assessment tools typically consist of an analytical checklist focusing on the content of the interview (assessing student knowledge) and a global checklist or rubric focusing on organization and communication skills such as verbal communication, nonverbal communication, demonstration of empathy, and a systematic approach. For the first cycle, we used a global assessment rubric adopted from a Canadian context that included these components. We quickly identified two cultural-related problems with this approach. First, up to 30% of our students wear a niqab and assessment of eye contact and nonverbal communication (i.e. facial expressions) was difficult to impossible. When assessment forms were returned, we found many “not applicable” scores, but also many scores were very low in comparison to other candidates (differing by 2–3 points on a 5-point scale, in favour for students not wearing niqabs). Secondly, best practice communication skills in one setting may not be the same in another.^6^ For example, in Qatar, Saudi Arabia and other Gulf nations, it may be considered rude or inappropriate to display extended eye contact, especially within mixed gender interactions.^7^ Patient-centered care is also largely a Western-based phenomenon that many patients with differing cultural backgrounds or religions may perceive as intrusive or not appropriate given the context of the interaction.^8^ Therefore, it is unclear if directly placing value on these items by including them on an assessment tool is appropriate for all contexts and settings.

#### Solutions

For our high-stakes summative assessment, we have adapted a 1-dimensional overall global scoring rubric to integrate components of structure, communication, and effectiveness of the interaction. This approach avoids breaking down components into categories that cannot be fairly assessed across all students within the cultural setting. The major drawback of this approach is that it does not allow for assessment and feedback pertaining to individual communication components. However, the variability in appropriateness of these components within our cultural context discredits attempts to break down communication assessment. Instead, assessors can be trained to grade/rate students according to the overall effectiveness of the interaction and how they adapted communication components to the patient’s own preferences.

### Challenge #2 – Standardized Actors

The use of actors within OSCEs aims to improve validity of the exam by minimizing error from standardized or real patients or other personnel. Standardized actors are trained to portray each case in the exact same way, with minimal to no deviation from the case and given script to achieve optimal reliability.^9^ However, we experienced great challenges both in recruiting and training of standardized actors that reflect the unique cultural characteristics of Qatar’s demographics. Most available actors are young to middle aged expatriates from Western countries and it is very difficult to recruit locals, Arab expatriates, and older actors. Many times, improvisations (i.e. wearing of local dress) are made based on available personnel, however this can negatively affect the face validity of the case depending on the cultural context of the interaction. For example, there are many cultural dynamics that may influence communication behaviours, especially when faced with angry or demanding patients. If the actor does not accurately reflect the appropriate demographic (i.e. Qatari), students may not perform as expected in practice and the overall intent of the station may be lost. Therefore, it is important students are assessed based on practice demographics in Qatar and efforts must be made to recruit actors matching these demographics.

Many contextual barriers exist regarding recruitment of sufficient numbers of actors. The population of Qatar, which is similar to other Gulf countries, is largely expatriate in nature. This results in a highly transient population that also has restrictions regarding employment under the country’s labour and sponsorship laws. Therefore, it is difficult to find and train appropriate personnel to portray needed roles. Additionally, acting groups (community or university based) and/or training programs are rare and cannot be relied upon to recruit suitable candidates.

#### Solutions

Recruitment in general was initiated by contacting other health professional programs in the country and gaining access to databases of standardized actors used. Unfortunately, this did not provide the necessary numbers and demographics needed for our high stakes exam. We then began recruiting using online message boards in Qatar, word of mouth, and through event planning agencies that hire performers such as dancers and other artistic talent. By doing so, we were able to recruit 50 actors in a three-month period. These actors participated in two training workshops prior to the exam. Subsequently, we hired many of these standardized actors for formative course-based assessments to further develop their expertise. It is from this pool that actors were selected to participate in the 2015 assessment cycle. A designated teaching assistant handled the recruitment and payment of the actors.

Matching demographics of Qatar’s population is still challenging yet efforts have been made to advertise and recruit from the local population through faculty and staff contacts. There are ongoing discussions with the Supreme Council of Health and other health professional programs to initiate a national standardized patient training and recruitment center, which should help to address this ongoing problem.

### Challenge #3 – Assessor Calibration

High stakes performance-based assessment encounters concerns regarding standardization of assessors and inter-rater reliability between assessors.^10^ This is especially true for judgments regarding communication behaviours and global skills.^11^ For our first cycle in 2014, we used both faculty and practicing pharmacist assessors and hosted a training session over four hours one month prior to the exam. This was supplemented by standardization during a dry run with all assessors and standardized patients the morning of the exam itself. Two assessors were present per station and inter-rater reliability was calculated for both analytical and global scoring. Reliability was found to be poor, especially for global performance (ICCs = 0.77 and 0.48, analytical and global components respectively).

#### Solutions

Assessor standardization or calibration is a strategy used to increase reliability between assessors.^10^ While there is a need to ensure assessments are valid, over-standardization may take away from assessor judgements and bias results in favour of examiner expectations. Therefore, perfect reliability (especially for global assessments) should not be the primary goal. However, we recognized a need to calibrate assessors after the first cycle and implemented strategies to do so. First, practicing pharmacist assessors were invited to take part in course-based formative OSCEs, which allowed for practice and experience evaluating student performance. Secondly, more comprehensive training was given, which incorporated large group discussion after observation of role-plays portraying differing levels of student performance. This strategy helped us identify differing assessor perspectives and work collaboratively with assessors through discussion to determine the student’s responses to an individual patient. Thirdly, assessors were given practice using analytical checklists of varying complexity and number of points (8–20 points), as reliability was the lowest in 2014 on checklists having more points and greater complexity. Finally, global assessment tools were modified to be simpler (as described above), in order to ensure they did not force assessors to evaluate something outside of cultural communication norms (i.e. facial expressions, eye contact). We believe these modifications were responsible for improved reliability observed in the 2015 cycle (ICCs = 0.88 and 0.61, analytical and global components respectively).

### Challenge #4 – Standard Setting with Angoff Method

We chose to use a modified Angoff method for establishing passing standards for each station in the 2014 OSCE.^12^ This method consists of a group of people reviewing the analytical checklist to come to consensus regarding the percentage of minimally competent graduates from our program who would successfully achieve each point. For example, 50% would translate into a point score of 0.5. All point scores are then added to determine the overall pass rate for the analytical checklist, which was merged with the standards set on the global assessment to obtain an overall station passing score. We completed this process with 6 groups of 5–6 participants consisting of both faculty and practicing pharmacists. Based on our collaborators’ experience in a Canadian context, we expected rich discussion and negotiation to occur for many of the checklist points. However, our process was completed in approximately 30 minutes per station, as very little discussion ensued. We believe this to be due to cultural factors that differ from Western settings. Specifically, we believe that the hierarchical nature of decision-making in the Middle East influenced this process and allowed one dominant group member (typically a faculty member) to direct the standards to be set in favour of his or her perceptions. This likely resulted in either inflation or deflation of passing standards. Consequently, we sensed this method may not be the best way to set standards in our context and we therefore began exploring alternatives.

#### Solutions

If the Angoff method^12^ for standard setting were to be repeated, we would plan for each group to have a facilitator that ensures every participant writes down or voices their opinion prior to discussion beginning. Participants should also be encouraged to be active in discussion and only change their beliefs if strong justification is given. These mechanisms should hopefully improve the validity of the process and result in a passing score representative of the entire group’s perceptions. However, there is potential that the facilitator might not be able to overcome the hierarchical and authoritarian cultural patterns.

In 2015, we therefore opted to use a different method for setting standards, the borderline regression method.^13^ This method was chosen based on a literature review of standard setting procedures and in consultation with colleagues from the University of Toronto. For this method, a borderline pass on the global assessment scoring system was deemed to be 3 out of 5. A scatterplot was then created based on scores for each station between analytical and global scores. A line of best fit was computed and used to determine the analytical score coinciding with the borderline global score. This score was then used as the analytical passing score. Although this method too may not be free from bias, we found it to be more practical than the Angoff method. We plan to use both methods and to study how any differences in passing scores affect overall passing rate of exam candidates and to further investigate cultural barriers to achieving effective standard setting through consensus procedures.

### Reflections on the Qatar-Canada Collaboration

Overall, our team in Qatar greatly benefited from the training and mentorship provided by Canadian collaborators for this national project in Qatar. It was an excellent example of a successful adoption and presentation of a rigorous assessment method into a new cultural context. Although challenges were experienced, they provided a rich learning experience for all participants from both countries. Upon further reflection, we believe the success of this project was largely due to the flexibility and understanding of the Canadian collaborators with respect to cultural and contextual considerations encountered throughout the process. It was also evident that the support from administration at Qatar University and the Supreme Council of Health in Qatar greatly motivated staff and volunteers to ensure a successful project. Finally, we believe the experience we shared with the Canadian collaborators will not only benefit Qatar’s practice but will also benefit future design of Canadian assessments, especially as Canadian cultural landscapes continually evolve and diversify.

### Summary

The challenges discussed in this paper are ones that programs must account for when attempting to export or develop high stakes OSCEs in new countries or settings. Many of these were unexpected and we hope that our experience will help others in designing and implementing assessments in their own context. The principles of assessment design and implementation we identified can be related elsewhere and will allow educators to recognize potential areas for improvement before embarking on similar assessment adoption. Specifically, programs must factor cultural communication norms and assessor cognition from a cross-cultural perspective when designing assessment procedures and evaluation tools. Additionally, particular attention must be paid towards ensuring standardized actor recruitment is appropriate for the cultural context, in order to adequately achieve the intended face validity of cases. Future studies should attempt to better understand how cultural and contextual factors influence the validity of OSCEs across borders and how intended best practices must be modified to fit the needs of local settings.
